# Extending import detection algorithms for concept import from two to three biomedical terminologies

**DOI:** 10.1186/s12911-020-01290-z

**Published:** 2020-12-15

**Authors:** Vipina K. Keloth, James Geller, Yan Chen, Julia Xu

**Affiliations:** 1 Department of Computer Science, New Jersey Institute of Technology, Newark, NJ 07102 USA; 2 Department of Computer Information Systems, Borough of Manhattan Community College, City University of New York, New York, NY 10007 USA; 3 JXU Consulting, 4343 Pine Blossom Tr., Houston, TX 77059 USA

**Keywords:** Terminologies, UMLS, Concept import, SNOMED CT, National cancer institute thesaurus, Density differences

## Abstract

**Background:**

While enrichment of terminologies can be achieved in different ways, filling gaps in the IS-A hierarchy backbone of a terminology appears especially promising. To avoid difficult manual inspection, we started a research program in 2014, investigating terminology densities, where the comparison of terminologies leads to the algorithmic discovery of potentially missing concepts in a target terminology. While candidate concepts have to be approved for import by an expert, the human effort is greatly reduced by algorithmic generation of candidates. In previous studies, a single source terminology was used with one target terminology.

**Methods:**

In this paper, we are extending the algorithmic detection of “candidate concepts for import” from one source terminology to two source terminologies used in tandem. We show that the combination of two source terminologies relative to one target terminology leads to the discovery of candidate concepts for import that could not be found with the same “reliability” when comparing one source terminology alone to the target terminology. We investigate which triples of UMLS terminologies can be gainfully used for the described purpose and how many candidate concepts can be found for each individual triple of terminologies.

**Results:**

The analysis revealed a specific configuration of concepts, overlapping two source and one target terminology, for which we coined the name “fire ladder” pattern. The three terminologies in this pattern are tied together by a kind of “transitivity.” We provide a quantitative analysis of the discovered fire ladder patterns and we report on the inter-rater agreement concerning the decision of importing candidate concepts from source terminologies into the target terminology. We algorithmically identified 55 instances of the fire ladder pattern and two domain experts agreed on import for 39 instances. In total, 48 concepts were approved by at least one expert. In addition, 105 import candidate concepts from a single source terminology into the target terminology were also detected, as a “beneficial side-effect” of this method, increasing the cardinality of the result.

**Conclusion:**

We showed that pairs of biomedical source terminologies can be transitively chained to suggest possible imports of concepts into a target terminology.

## Background

The Metathesaurus of the Unified Medical Language System (UMLS) [1] is a large biomedical thesaurus of concepts from 211 source terminologies (2019 AB release) in 25 different languages. It is organized by linking all names for the same concept under a Concept Unique Identifier (CUI). The Metathesaurus identifies the different relationships between the concepts and also preserves the concept names, concept IDs and the relationships between the concepts in each source terminology. The terminologies in the UMLS differ widely in their domains and application areas. For example, the Logical Observation Identifiers Names and Codes terminology (LOINC^®^) [2] is a terminology for the standardized exchange of laboratory data, while the Gene Ontology (GO) [3] describes gene products in terms of their associated biological processes, cellular components, and molecular functions. However, there are many terminologies that cover multiple domains. For example, the SNOMED CT [4] provides the core general terminology for Electronic Health Records (EHRs) by organizing concepts into hierarchies (*Body structure*, *Clinical finding*, *Specimen*, etc.) and has over 350,000 unique, active concepts. As a result, there is substantial overlap in the conceptual content between the SNOMED CT and several other terminologies.

**Fig. 1:**
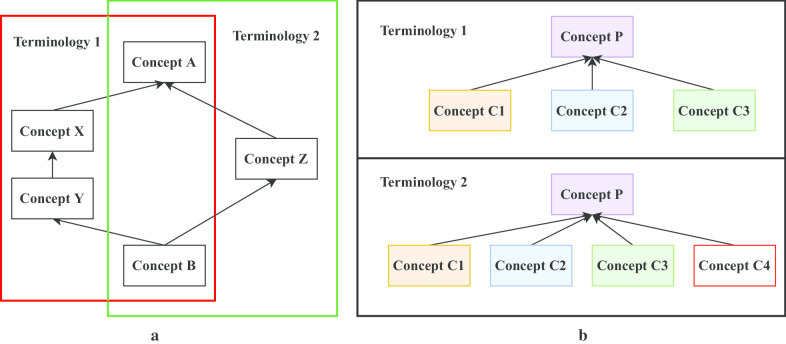
**a** Vertical density difference (“diamond”), **b** horizontal density difference. All arrows indicate IS-A relationships

Previously, we have observed that when pairs of terminologies in the UMLS have overlap in their conceptual contents, they nevertheless may have notable differences with respect to their vertical and horizontal densities [5–8]. A vertical density difference occurs when *“IS-A”*/*concept paths* of different lengths exist in two terminologies that are constrained by begin/end concepts that are identical in both the terminologies (Fig. 1a). We use the term “density” following Rector et al. [9]. The resulting topological pattern was referred to as a *diamond* [10]. A horizontal density difference arises out of the fact that the same concept in two different terminologies may have different sets of children in each terminology (Fig. 1b) [8]. These differences led to several questions like (a) are some concepts missing from one terminology and if so could these missing concepts be imported into that terminology, (b) are these differences the result of some error in one or both of the terminologies, or (c) are these differences due to concepts in one terminology being synonyms to concepts in the other terminology? Detailed investigations of all such cases were performed in prior research and the results were analyzed by domain experts [5] who confirmed many possible cases of concept import, which in turn results in terminology enrichment.

This paper explores whether topological patterns analog to diamonds (Fig. 1a) exist when considering more than two terminologies at a time and whether the resulting patterns suggest possible import of concepts from one terminology into another. While such suggestions should be derived algorithmically, the final decision on an import is always made by a human expert.

**Fig. 2:**
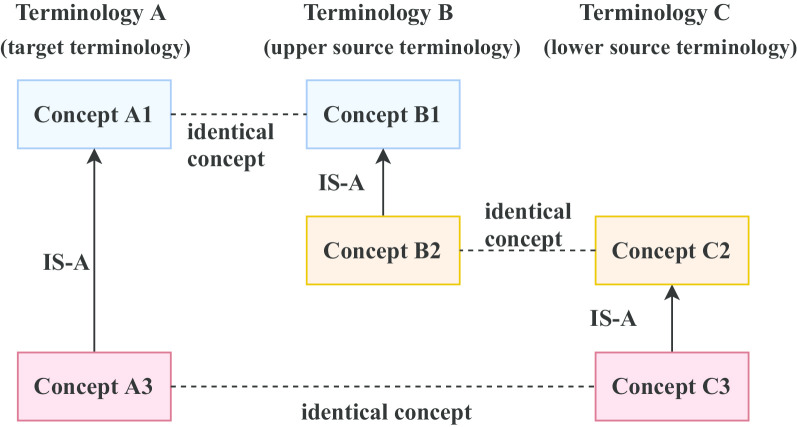
An abstract fire ladder pattern

One of the possible extensions of the study on vertical density differences involves the concepts in three terminologies as shown in Fig. 2. Consider three terminologies A, B, and C. The concept A1 in terminology A has a child concept A3, the concept B1 in terminology B has a child B2, and the concept C2 in terminology C has a child C3. The concepts A1 and B1 are identical by means of having the same UMLS CUI. Similarly, the concepts B2 and C2 are identical, and so are A3 and C3. It should also be noted that the concept C3 (= A3) does not exist anywhere in terminology B, the concept B2 (= C2) does not exist anywhere in terminology A, and the concept A1 (= B1) does not exist anywhere in terminology C. Looking only at A1, B1, B2, C2, and ignoring that the connections between them are of two different kinds (IS-A versus identity) this identifies a kind of transitivity (Fig. 2) [11].

Because we are chaining together two vertical patterns to jointly achieve a “higher reach” we are reminded of an extensible ladder as they are carried by fire trucks. Thus, we will refer to the pattern in Fig. 2 as the *fire ladder pattern* in contrast to the diamond patterns that we have investigated previously for vertical density (Fig. 1a). We refer to A as the target terminology, to B as the “upper source terminology,” and to C as the “lower source terminology.” The primary questions that arise from Fig. 2 are whether B2 (= C2) should be proposed for import into terminology A, and whether C3 should be recommended for import into terminology B.

Thus, in this paper, we quantitatively explore the fire ladder patterns formed by the concepts from 10 different terminologies in the UMLS Metathesaurus. We developed an algorithm that suggests concepts that could potentially be imported into another terminology. We also had two domain experts review the suggestions made by the algorithm for deciding whether the concepts should be imported or not. We note that one other import is suggested by Fig. 2, which we will elaborate on in the Discussion Section.

### UMLS

The UMLS Metathesaurus is a large, multi-purpose, and multi-lingual repository of biomedical and health-related terminologies. The Metathesaurus maintains information about concepts, their synonyms and the relationships among them. Similar terms from different source terminologies are organized into a concept that is identified by a Concept Unique Identifier (CUI), e.g. C0018799 stands for *Heart diseases*. The concepts are linked to each other by means of different relationships identified by a Relationship Unique Identifier (RUI) [12]. All relationships in the Metathesaurus are given a general label (REL), describing the nature of the relationship like *Child of*, *Broader*, *Qualifier of*, etc. Furthermore, about one quarter of the relationships carry an additional label (RELA—Relationship Attribute). Labels are obtained from each source terminology and include, e.g., *IS-A, component_of, part_of*, etc. For the experiments described in this paper, we used the 2018 AB release of the UMLS with a focus on PAR (*Parent of*) relationships with an additional *inverse_isa* Relationship Attribute, together corresponding to what is commonly known as an IS-A link.

### Related work

#### Density differences

In prior work, we utilized the structure of the UMLS to identify the vertical and horizontal density differences for concepts from pairs of terminologies to find potential concepts for import that could help in achieving semantic harmonization among terminologies. He et al. [5] defined “structurally congruent concepts” and interpreted them in different ways including alternative classifications, synonyms, and errors in a terminology. A definition of *alternative classifications* is beyond the scope of this paper. This idea was later extended to identify topological patterns called trapezoids or diamonds arising from the vertical density differences, to import missing concepts into the SNOMED CT and National Cancer Institute Thesaurus (NCIt) [6, 7, 13]. A quantitative analysis of the difficulty in importing the pattern-based concepts was also performed [10, 14]. We subsequently proposed a metric for identifying likely cases of alternative classifications using horizontal density differences [15].

Sun and Zhang’s method for identifying granularity differences and similarities between biomedical ontologies uses a rule-based approach, where a rule inference engine constructs rules to explore structural incompatibilities [16, 17]. Luo et al. [18] proposed “parallel concept sets (PCS)” to identify the granularity balance of IS-A and *part_of* relationships within one biomedical ontology, while we always worked with pairs of ontologies.

#### Ontology matching/alignment

Ontology alignment is the process of finding semantic correspondences between different ontologies [19–21]. The mappings are usually based on concept names, definitions, and relationships between concepts in the ontologies. Most research in this field focuses on identifying 1:1 correspondences between concepts in different ontologies [22, 23]. For example, Bodenreider et al. [24] reported alignment of mouse and human anatomies by investigating the NCIt (for the human anatomy) and the Adult Mouse Anatomical Dictionary. Certain complex correspondences (*1:n* and *m:n*) [25] and ternary compound alignments [26] were also reported in targeted studies.

For applications involving pairs of (or, less often, multiple) ontologies, the alignment/matching techniques help ensuring interoperability by establishing semantic mappings between the ontologies. On the other hand, our techniques, involving density differences, help with identifying concepts that are potentially missing in one ontology. Those concepts could be imported from one ontology into another whenever a human expert agrees.

#### Ontology quality assurance and semantic enrichment

Quality assurance is an important part of the ontology life cycle and has been widely studied [27–31]. Different studies have focused on different aspects such as structural relationships (e.g. IS-A, part-of), semantic type assignments, and different methodologies (e.g. lattice-based [32], abstraction-network-based [33] etc.). Several studies have focused on lattice-based structural auditing, as the hierarchical structure of an ontology is expected to be a lattice, as a criterion for its well-formedness [32, 34]. Zhu et al. [35] compared the subsumption relationship between FMA and SNOMED CT’s *Body Structure* hierarchy, to understand structural disparities and analyze the non-lattice fragments in SNOMED CT. Zhang and Bodenreider [32] proposed a lattice-based approach for exhaustive auditing of SNOMED CT, while Zhu et al. [36] used concept lattices for evaluating the semantic completeness of SNOMED CT.

While most studies focused on auditing a single ontology, Cui [37] proposed a cross-ontology method for identifying inconsistencies and errors across multiple ontologies in the UMLS. Even though the direct goal of our methods [7, 8, 15], based on density differences, was not quality assurance, as a by product these methods have identified inconsistencies and errors in different ontologies. On the other hand, Zhang and Bodenreider [32] reported that lattice-based studies for auditing ontologies are in turn effective in identifying potentially missing precoordinated concepts in SNOMED CT for semantic enrichment. While our methods identify already existing concepts in other ontologies that are missing in the target ontology, the lattice-based approaches identify precoordinated concepts which, when introduced, will make non-lattice fragments into lattice-conforming structures that are ontologically well-formed.

## Methods

The fire ladder pattern is formed by concepts having a PAR relationship with an inverse_isa Relationship Attribute, which denotes in the UMLS what was called “IS-A” in previous sections. We selected from the UMLS all the terminologies in English that use IS-A relationships to form a hierarchy (more precisely: a Directed Acyclic Graph). This resulted in 12 terminologies out of the 207 source terminologies in the 2018 AB release of the UMLS. For the studies reported in this paper, two terminologies, the Veterinary Extension to SNOMED CT (SNOMEDCT_VET) and the University of Washington Digital Anatomist (UWDA) were excluded as they are subsets of two other terminologies. The remaining 10 terminologies are the SNOMED CT, NCIt, MEDCIN, Anatomical Therapeutic Chemical Classification System (ATC), Medical Entities Dictionary (CPM), Current Procedural Terminology (CPT), Foundational Model of Anatomy Ontology (FMA), Gene Ontology (GO), Human Phenotype Ontology (HPO), and Universal Medical Device Nomenclature System (UMD). Below we will refer to them simply as T_1_, T_2_, …, T_10._ We then proceeded to develop an algorithm that detects concepts from two different terminologies for possible import into a third terminology when the three form a fire ladder pattern.

### Algorithm

The algorithm has two parts. FIRE_LADDER is the top level algorithm. It generates the set PT of all distinct triples of terminologies taken from the set T = {T_1_, T_2_, …, T_10_}, i.e., PT = {<T_1_, T_2_, T_3_>, <T_1_, T_2_, T_4_>, …, <T_8_, T_9_, T_10_>}. Because one of these three terminologies is designated the target terminology, the second is the “upper source” and the third is the “lower source,” (Fig. 2) <T_1_, T_2_, T_3_> is distinct from <T_1_, T_3_, T_2_>, etc. Thus, PT is really the set of all permutations [38] of three terminologies taken from 10 terminologies. Therefore, there are 720 triples in PT, according to the formula

1 $$P \, \left( {n, \, k} \right) = n! \div \left( {n \, {-} \, k} \right)!$$

where *k* = 3 and *n* = 10.

The second part of the algorithm, named FIRE_LADDER_SUB, takes two inputs, namely ontDAG and the set PT generated by FIRE_LADDER. The parameter ontDAG is a Python dictionary (key-value pairs) structure (the details of which are described in [8, 15]), where each terminology has a sub-dictionary with a concept as key and a list of all its parents and a list of all its children as values. (This approach can be implemented in any language with a hash table mechanism.) For example, the terminology CPT has a sub-dictionary with 13,482 concepts each maintaining a list of its parents and its children. The presence of cycles and self-loops of IS-A links in the UMLS can result in inconsistencies [39, 40]. While creating ontDAG, cycles were detected and removed [41]. For removing the cycles we used an adaptation of the "naïve" (by their own appellation) approach to eliminating cycles by Mougin and Bodenreider [41]. This approach performs a depth-first search of the Metathesaurus graph and marks nodes as visited to detect loops. We adapted this approach by using only concepts that participate in an IS-A relationship (PAR, inverse_isa) in the 10 terminologies used in our study, instead of all the hierarchical relationships in the Metathesaurus, and also limited the maximum depth to five levels instead of the 50 levels of Mougin and Bodenreider [41], as the patterns described in this paper would never go beyond five levels for any concept.The pseudocode of FIRE_LADDER_SUB is given below.
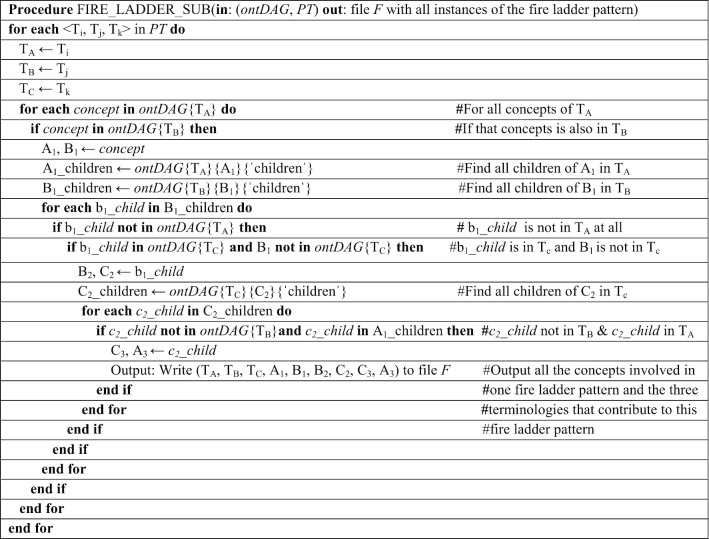


**Fig. 3:**
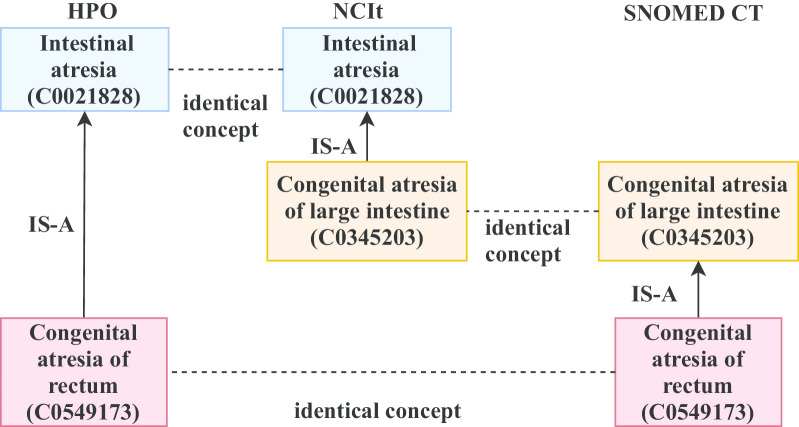
An example of a fire ladder pattern with the terminologies HPO, NCIt, and SNOMED CT. The UMLS CUIs of the concepts are provided inside the parentheses

The algorithm outputs a file with information about sets of concepts that form a fire ladder pattern and the three terminologies each concept set is derived from. The total time to execute the script corresponding to the above algorithms and to generate the output file was approximately 22 s on an Intel(R) Core i5 CPU with four cores and ~ 2.4 GHz clock speed. An example involving the terminologies HPO, NCIt and the SNOMED CT is shown in Fig. 3.

Figure 3 is based on UMLS Concept IDs (CUIs, starting with the letter C). These concepts will have different ID numbers in the three source terminologies. Furthermore, a concept might have a different preferred term in the UMLS versus in a source terminology. However, the shared UMLS CUI guarantees that concepts that appear different in the native browsers of different source terminologies are in reality the same concept.

Thus, in Fig. 3, *Intestinal atresia* has the unique ID HP:0011100 in HPO and the code C84790 in NCIt. *Large Intestine Atresia* (NCIt ID: C98827) is the preferred name for *Congenital atresia of large intestine* in NCIt, whereas SNOMED CT uses the preferred term *Atresia of large intestine* (SNOMED CT ID: 204711007). The preferred term for *Congenital atresia of rectum* in HPO is *Rectal atresia* (HP:0025023). SNOMED CT uses the term *Congenital atresia of rectum* (91375006). It should be noted that HPO has a term *Colonic atresia* (HP:0010448) with a synonym *Large intestinal atresia*, which is listed as the child of *Intestinal atresia* in this ontology. In the UMLS *Colonic atresia* has the CUI C0266190, whereas the CUI for *Large intestinal atresia* is C0345203, showing that the UMLS considers them as two different concepts.

### Evaluation

We created two data sets (Data Set 1 and Data Set 2) from the fire-ladder pattern (Fig. 3) to be reviewed by our two domain experts (YC and JX). YC has training in sports medicine and a PhD in Computer Science with a concentration in Medical Informatics. JX has an MD degree and MS and PhD degrees in Medical Informatics. Both have years of experience and many publications in medical ontologies/terminologies. Data Set 1 corresponds to the enrichment of terminology A by importing B2. For this data set, we provided the domain experts with the names of the three terminologies (A, B and C) and also the concepts A1 (= B1), B2 (= C2), and C3 (= A3) and asked for their judgement on whether the concept B2 should be imported into terminology A as the child of A1 and parent of A3.

It should be noted that the fire ladder pattern supports another possible import resulting from the horizontal density difference between the terminologies B and C. Thus, we also asked the domain experts about their judgement on importing C3 (= A3) into terminology B as a child of B2. Accordingly, for this Data Set 2 we provided the domain experts with the names of the terminologies (B and C) and the concepts B2 and C3. For this import, B would become the target terminology and C would simply be the source terminology without qualification as upper or lower. This kind of import would be similar to our previous work on horizontal density differences [8]. However, a larger number of ontology combinations are investigated in this paper.

The review of Data Set 1 was done in two phases. In the first phase, along with the decision on whether a concept should be imported or not, we also asked the domain experts to provide the reasons behind their judgement. Once we received the results of the first phase from both of our domain experts, we initiated another round of reviews limited to those patterns on which the domain experts disagreed with each other. In this phase, we showed both of them the reasons behind each other’s decisions. This resulted in only one change to the data for Data Set 1, increasing the metric of agreement minimally. We computed inter-rater agreement based on Krippendorf’s α and Cohen Kappa.

## Results

We found 26 triples for the 10 terminologies analyzed, forming fire ladder patterns out of the possible 720 triples according to Eq. (1). For Data Set 1, we identified 55 *distinct* B2 concepts (using our algorithms) that were reviewed by the experts for import into terminology A. There were two cases (in addition to the 55 mentioned above) in which the same triple of concepts (A1, B2, C3) was formed by different permutations of terminologies. For example, A1: *Rhabdomyoma*, B2: *Cardiac rhabdomyoma*, C3: *Congenital rhabdomyoma of heart* was formed by the triple <SNOMED CT, NCIt, MEDCIN> and the triple <SNOMED CT, HPO, MEDCIN>. Since the target terminology is the same (SNOMED CT in this case), these two permutations were considered together for Data Set 1, yielding a total of 55 distinct B2 concepts for a total of 57 fire ladder patterns discovered. Table 1 shows each triple of terminologies and the number of fire ladder patterns formed by the permutations of these terminologies. There were 18 instances formed by permutations of {SNOMED CT, MEDCIN, CPT} and another 17 instances by permutations of {SNOMED CT, NCIt, MEDCIN} accounting for more than half of the candidate concepts. It should be noted that columns one and three in Table 1 represent permutations of triples of terminologies and not a single triple. For example, the triples <HPO, SNOMED CT, NCIt> and <HPO, NCIt, SNOMED CT> contributed two fire ladder patterns each to get the four patterns listed in the third row of Table 1.

**Table 1:** Triples of terminologies and the number of fire ladder patterns formed by permutations of each triple

All permutations of triple of terminologies	Number of fire ladder patterns	All permutations of triple of terminologies	Number of fire ladder patterns
CPT, SNOMED CT, MEDCIN	18	UMD, SNOMED CT, NCIt	2
NCIt, SNOMED CT, MEDCIN,	17	MEDCIN, ATC, NCIt	2
HPO, SNOMED CT, NCIt	4	SNOMED CT, HPO, MEDCIN	2
CPT, NCIt, MEDCIN	3	CPM, SNOMED CT, NCIt	1
FMA, SNOMED CT, NCIt	3	HPO, SNOMED CT, MEDCIN	1
SNOMED CT, CPM, MEDCIN	3	SNOMED CT, GO, FMA	1

Out of the 55 concepts suggested for import by our algorithm for Data Set 1, one domain expert agreed on importing 42 concepts (76.3%) and the other agreed on 45 concepts (81.8%) (Table 2). The two domain experts agreed in their decisions regarding 39 out of 55 concepts (71%). We calculated the inter-rater agreement using Krippendorff’s α score and Cohen Kappa and obtained a value of 0.51 and 0.507 respectively. Examples of some fire ladder patterns are shown in Table 3. All fire ladder patterns obtained are listed in the Additional file 1.

**Table 2:** Details of the domain experts’ decisions regarding importing the concepts out of 55 suggestions made by the algorithm

Domain expert 1		Domain expert 2		Two domain experts		
Recommends import	Recommends non-import	Recommends import	Recommends non-import	Both recommend import	Both recommend non-import	One Expert for import one against
42	13	45	10	39	7	9

**Table 3:** Examples of fire ladder patterns. The concept B2 was agreed on by our experts to be imported into terminology A as a child of A1 and as a parent of A3

Term. A	Term. B	Term. C	Concept A1	Concept B2	Concept A3
SNOMED CT	MEDCIN	CPT	Drug measurement	Therapeutic drug assays	Theophylline assay
NCIt	MEDCIN	SNOMED CT	Urologic surgical procedures	Operation on urethra	Urethrostomy
HPO	SNOMED CT	NCIt	Adrenal gland hypofunction	Adrenal cortical hypofunction	Secondary adrenal insufficiency

For Data Set 2, we identified 105 distinct pairs of concepts (B2, C3) in terminologies B and C. We observed that for one concept B2, there were several concepts in the position of C3. For instance, for the fire ladder pattern formed by A1: *Tract of spinal cord*, B2: *Descending spinal cord tract* we observed two different C3s namely *Structure of medial reticulospinal tract* and *Structure of lateral reticulospinal tract*. While for Data Set 1 each algorithmic suggestion would potentially result in importing one concept into terminology A, for Data Set 2 we have two potential imports into terminology B in this example.

The domain expert (JX) agreed to import 98 concepts out of 105 concepts (93.33%). Examples are shown in Table 4.

**Table 4:** Examples from Data Set 2. The concept C3 was agreed to be imported into terminology B as the child of concept B2

Term. B	Term. C	Concept B2	Concept C3
MEDCIN	NCIt	Vital signs measurements	Heart rate
HPO	MEDCIN	Cardiac rhabdomyoma	Congenital rhabdomyoma of heart
NCIt	SNOMED CT	Colon carcinoma	Carcinoma of descending colon
ATC	NCIt	Thyroid hormones	Levothyroxine sodium
GO	FMA	Region of chromosome	Short arm of chromosome

We performed an error analysis for cases in which the domain experts did not recommend algorithmically determined candidate concepts for import. One example from Data Set 1 consists of the fire ladder pattern formed by A1: *Metastatic Neoplasm*, B2: *Secondary Neoplasm* and C3: *Metastasis to digestive organs*. According to our domain experts, A1 and B2 are sufficiently close to each other to be considered as synonyms. For Data Set 2, the concept *anterior radial head dislocation* was not imported as the child of *Congenital dislocation of radial head,* because the former concept is not necessarily congenital.

## Discussion

It has been argued in the biomedical ontology community that bigger is not necessarily better. However, we observe that many major ontologies and terminologies have been growing monotonically for the past several years. That means that every release in recent years has contained more concepts than the previous release. This has been the case for the SNOMED CT, with more than 50,000 concepts added in the past five years [42]. Similarly, more than 40,000 concepts have been added to NCIt [43]. Our argument is that if ontologies are demonstrably extended “anyway,” they should be extended in a systematic process that leads to more harmonization between major, widely used ontologies in the field. Furthermore, the “damage” for a medical user not finding a desired concept is bigger than for another user having to ignore an additional concept.

The question of the right degree of pre-coordination has been discussed previously in the literature, e.g., [32]. On one hand, the difficult task of post-coordinating concepts should not be left to the users, who are likely not experienced and knowledgeable about ontologies. On the other hand, creating a large number of pre-coordinated concepts increases both the effort of the curator and the search effort of the user, because these concepts are "cluttering up" the ontology. Finding the right balance between too much pre-coordination and too little pre-coordination is difficult.

In our previous extensive experience in Quality Assurance of Biomedical Ontologies we have found that curators often reject the inclusion of new concepts, not because they would make the granularity too high, but because they feel there is no use case for those concepts and their customers would not need them.

In our case, one could aim for a balanced degree of granularity. For this, the path length from the root to a leaf within a specific hierarchy could be used as a stand in for a measure of granularity. Thus, if our algorithm proposes import of a concept into a path that consists of a below average number of concepts between the root and the leaf node, this could be encouraged, while the opposite would be the case for paths that are already very long and detailed. The path length comparison would need to be done within a specific hierarchy or even subhierarchy, because different subject areas will favor a more detailed or less detailed breakdown of the available knowledge. However, details of this analysis need to remain for future work.

It is important to stress the contribution of using two source terminologies in tandem, which is a novel method reported for the first time in this paper. In Data Set 1, we can be quite confident that a suggested candidate for import is correct, because it is constrained from above and below. While there have been cases [7] where candidates were constrained from above and below by a single source terminology, this was not possible for the 55 candidate concepts that there were discovered in this paper. For Data Set 2, a candidate concept for import is only constrained from above, similar to our previous work [8], which is a weaker indication that an import is desirable.

One can think of a third possible case of import based on Fig. 2, which is importing B1 (= A1) into terminology C as a parent of C2. However, this presents another question as to how to find a parent for the new C1, given that we should have a path from every concept to the root of its terminology, following design standards in the field of ontologies and terminologies.

The question arises whether transitive patterns can be constructed for four terminologies at a time. We performed research on this question and were not able to identify any such patterns within the UMLS. Another question, to be explored in the future, is whether the import of a concept could lead to the subsequent discovery of new vertical density differences. Thus, after importing B2 into A (Fig. 2), A1 and B2 together could form the right side of a new diamond (Fig. 1a) with a fourth terminology.

There is one more approach to extend the set of density-based methods for discovering candidate concepts for import. For this, we have to refer back to Fig. 2. There, we assumed that B2 is a child of B1. However, it is possible that B1 and B2 together define a path with one or more intermediate concepts between them. Let us assume that there is exactly one such intermediate concept that we will name B1.5. In that case, the fire ladder pattern of Fig. 2 would suggest the import of both B2 and B1.5 into the terminology A. This approach can also be extended for importing concepts from terminology C into terminology B, by extending the length of the path between C2 and C3 and adding intermediate concepts such as "C2.5" between them. Investigating this kind of pattern requires a more complicated algorithmic approach and is left for future work.

The number of proposed imports in this paper is relatively smaller than in our previous papers. For example, Keloth et al. [8] showed that 7099 concepts were algorithmically suggested for import into SNOMED CT. The smaller cardinality of results in this paper reflects a classical instance of the law of diminishing returns [44]. The “low hanging fruit” had already been harvested in previous papers, and in this paper, a more powerful method had to be applied for a marginal increase in results. Thus, this paper should not be seen in isolation, but as one of the final building blocks of a multi-year research program that had started in 2014 [5] with the goal of informing the content of one terminology by one or more other terminologies linked together by the UMLS Metathesaurus.

## Conclusions

In this paper, we proposed a novel topological pattern called *fire ladder* and an algorithm to discover such patterns in triples of terminologies to help identify potentially missing concepts in 10 UMLS terminologies. This pattern consists of two source terminologies used in tandem and one target terminology. We found 55 instances of fire ladder patterns, out of which two experts agreed on 39 instances of concept imports. For 48 (= 39 + 9; 87%) instances at least one expert agreed that the algorithm reported a viable import. Furthermore, the import of 98 additional concepts out of 105 algorithmically discovered candidate concepts was recommended, based on one source terminology and one target terminology.

## Supplementary information

**Additional file 1.** Table listing all fire ladder patterns obtained.

## Data Availability

The dataset supporting the conclusions of this article is available in the UMLS Knowledge Sources repository [https://www.nlm.nih.gov/research/umls/licensedcontent/umlsknowledgesources.html].

## Abbreviations

UMLS: Unified medical language system
CUI: Concept unique identifier
GO: Gene ontology
EHRs: Electronic health records
PAR: Parent of
NCIt: National Cancer Institute Thesaurus
CPT: Current procedural terminology
FMA: Foundational model of anatomy
HPO: Human phenotype ontology
ATC: Anatomical therapeutic chemical classification system
CPM: Medical entities dictionary
UMD: Universal medical device nomenclature system
