# Supplementary material for: Extending import detection algorithms for concept import from two to three biomedical terminologies
Source: BMC Med Inform Decis Mak. 2020 Dec 15;20(Suppl 10):272. doi: 10.1186/s12911-020-01290-z (PMC7737255; doi:10.1186/s12911-020-01290-z)
Supplement: Supplementary file 1 — Additional file 1. Table listing all fire ladder patterns obtained. [file 12911_2020_1290_MOESM1_ESM.pdf]

**Table listing all fire ladder patterns obtained**

| Term. A | Term. B | Term. C | A1 | B2 | C3 |
|---|---|---|---|---|---|
| SNOMED CT | NCI | MEDCIN | C0011923 : Diagnostic Imaging | C1881134 : Imaging studies | C0016313 : Fluorescein Angiography |
| SNOMED CT | NCI | MEDCIN | C0031150 : Laparoscopy | C1883297 : laparoscopy (therapeutic) | C0521267 : Laparoscopic adrenalectomy (procedure) |
| SNOMED CT | NCI | MEDCIN | C0027543 : Avascular necrosis of bone | C0745048 : asceptic necrosis of humerus | C0410481 : Avascular necrosis of the head of humerus |
| SNOMED CT | NCI, HPO | MEDCIN | C0035411 : Rhabdomyoma | C1332852 : Cardiac rhabdomyoma | C0265875 : Congenital rhabdomyoma of heart |
| SNOMED CT | NCI | MEDCIN | C0346770 : Malignant tumor of epidermal appendage | C1382026 : Malignant Sebaceous Neoplasm | C0346771 : Primary malignant neoplasm of sebaceous gland |
| SNOMED CT | MEDCIN | CPM | C0002771 : analgesics | C0027415 : Narcotics | C0376196 : Opiates |
| SNOMED CT | MEDCIN | NCI | C0518766 : Vital signs | C2963216 : Vital signs measurements | C0018810 : heart rate |
| SNOMED CT | MEDCIN | CPT | C1261153 : Drug measurement | C0374834 : therapeutic drug assays | C0006950 : Carbamazepine measurement, C0039773 : Theophylline Assay, C0202348 : Caffeine measurement, C0202381 : Ethosuximide measurement, C0202404 : Lidocaine measurement, C0202454 : Phenytoin measurement, |

| | | | | | |
|---|---|---|---|---|---|
| | | | | | C0202456 : Primidone measurement, C0202457 : Procainamide measurement, C0337448 : Cyclosporine measurement, C0337449 : Digoxin measurement, C0337452 : Lithium measurement, C0337453 : Quinidine measurement, C0519826 : Assay for Tacrolimus, C0519827 : Assay for Topiramate, C0524166 : Lamotrigine measurement, C0524238 : Phenobarbital measurement, C0919241 : Assay of haloperidol, C1266759 : Tiagabine measurement, C1618097 : Sirolimus measurement |
| SNOMED CT | MEDCIN | CPM | C0042014 : Urinalysis | C1254471 : Urine Panels | C0202510 : Urinalysis, Bacteriuria screen |
| SNOMED CT | MEDCIN | CPT | C1293305 : Foot repair | C2224835 : Midfoot capsulotomy | C0188689 : Capsulotomy of midfoot with |

| | | | | | |
|---|---|---|---|---|---|
| | | | | | tendon lengthening |
| SNOMED CT | GO | FMA | C0729606 : Chromosome structures | C1953345 : Region of chromosome | C0007709 : Centromere, C0008545 : Chromatids, C0457309 : short arm of chromosome, C1276871 : long arm of chromosome |
| CPM | NCI | SNOMED CT | C1254349 : amino acid, peptide or protein | C0030956 : Peptides | C0030520 : Parathyroid hormone, C0037663 : Growth hormone, C0376180 : Gastrin |
| NCI | SNOMED CT | MEDCIN | C1333803 : Gastrointestinal System finding | C0426576 : Gastrointestinal symptom | C0037089 : Signs and symptoms, Digestive |
| NCI | SNOMED CT | MEDCIN | C0017416 : Genital Neoplasms, Female | C1263788 : Neoplasm of parametrium | C1519870 : Neoplasm of uterine ligament |
| NCI | SNOMED CT | MEDCIN | C0729555 : Infection of digestive system | C0178238 : Intestinal infectious disease (disorder) | C1698036 : Small intestinal infection, C1699694 : Cecal infection, C2700620 : anorectal infection |
| NCI | SNOMED CT | MEDCIN | C0038454 : Cerebrovascular accident | C1263853 : Paralytic stroke | C0553692 : Brain hemorrhage |
| NCI | MEDCIN | ATC | C0019932 : Hormones | C0279175 : Thyroid preparations | C0040135 : Thyroid hormones |
| NCI | MEDCIN | SNOMED CT | C0032284 : Pneumonectomy | C2082610 : Total pneumonectomy | C0396585 : sleeve lobectomy |
| NCI | MEDCIN | SNOMED CT | C0038913 : Urologic | C0194546 : Operation on urethra | C0194560 : Urethrostomy (procedure), |

| | | | | | |
|---|---|---|---|---|---|
| | | | surgical Procedures | | C2959803 : Cystourethrectomy |
| NCI | MEDCIN | SNOMED CT | C0038913 : Urologic surgical Procedures | C0869844 : Procedures on Ureter | C0041963 : Ureterostomy procedure |
| NCI | CPT | MEDCIN | C0201952 : chloride measurement | C0391938 : Blood chloride level measurement | C1317978 : Serum chloride measurement |
| MEDCIN | SNOMED CT | NCI | C0304509 : Inotropic agent | C0031638 : Phosphodiesterase Inhibitors | C0148345 : vesnarinone |
| MEDCIN | SNOMED CT | CPT | C0869813 : Procedures on Appendix | C0192819 : Operation on appendix | C2191406 : Appendiceal laparoscopy |
| MEDCIN | SNOMED CT | NCI | C0042866 : Vitamin D | C1520059 : Vitamin D analog | C0043872 : maxacalcitol |
| MEDCIN | SNOMED CT | HPO | C0434609 : Dislocation of radial head | C0265563 : Congenital dislocation of radial head | C1867398 : Radial Heads, Posterior Dislocation Of, C2674451 : anterior radial head dislocation |
| MEDCIN | CPM | SNOMED CT | C0027415 : Narcotics | C0376196 : Opiates | C2001271 : tapentadol, C0724655 : opium tincture |
| MEDCIN | NCI | SNOMED CT | C0009402 : Colorectal carcinoma | C0699790 : Colon carcinoma | C0559002 : Carcinoma of descending colon, C0559078 : Carcinoma of ascending colon, C0589631 : Carcinoma of transverse colon |
| MEDCIN | NCI | CPT | C0193388 : Biopsy of liver (procedure) | C0581276 : Needle biopsy of liver | C1261294 : Percutaneous needle biopsy liver |

| | | | | | |
|---|---|---|---|---|---|
| MEDCIN | NCI | SNOMED CT | C2939420 : Metastatic Neoplasm | C2939419 : Secondary Neoplasm | C0555276 : Metastasis to digestive organs |
| MEDCIN | ATC | NCI | C0279175 : Thyroid preparations | C0040135 : Thyroid hormones | C0079226 : Dextrothyroxine sodium, C0079691 : Levothyroxine sodium, C0546882 : liothyronine sodium, C0936106 : liotrix |
| MEDCIN | CPT | SNOMED CT | C0374834 : therapeutic drug assays | C0002500 : Amikacin Assay | C1278112 : Serum amikacin measurement |
| MEDCIN | CPT | SNOMED CT | C0374834 : therapeutic drug assays | C0006950 : Carbamazepine measurement | C0428223 : Carbamazepine blood level measurement |
| MEDCIN | CPT | SNOMED CT | C0374834 : therapeutic drug assays | C0202381 : Ethosuximide measurement | C0428226 : Serum ethosuximide measurement |
| MEDCIN | CPT | SNOMED CT | C0374834 : therapeutic drug assays | C0202456 : Primidone measurement | C0428240 : Primidone blood measurement |
| MEDCIN | CPT | SNOMED CT | C0374834 : therapeutic drug assays | C0337449 : Digoxin measurement | C0428224 : Digoxin blood measurement |
| MEDCIN | CPT | SNOMED CT | C0374834 : therapeutic drug assays | C0337452 : Lithium measurement | C0428230 : Lithium blood measurement |
| MEDCIN | CPT | SNOMED CT | C0374834 : therapeutic drug assays | C0519826 : Assay for Tacrolimus | C1271702 : Blood tacrolimus level |
| MEDCIN | CPT | SNOMED CT | C0374834 : therapeutic drug assays | C0519827 : assay for topiramate | C1271826 : Serum topiramate level (procedure) |
| MEDCIN | CPT | SNOMED CT | C0374834 : therapeutic drug assays | C0524166 : Lamotrigine measurement | C1278122 : Serum lamotrigine measurement |

| | | | | | |
|---|---|---|---|---|---|
| MEDCIN | CPT | SNOMED CT | C0374834 : therapeutic drug assays | C1618097 : Sirolimus measurement | C1445979 : Blood sirolimus measurement |
| FMA | NCI | SNOMED CT | C0935624 : Capillary vessel | C0006901 : Blood capillaries | C0221919 : structure of capillary of skin |
| FMA | NCI | SNOMED CT | C1519456 : Tract of spinal cord | C1282010 : ascending spinal cord tract | C0228585 : Structure of lateral spinothalamic tract, C0228586 : Spinotectal tract of spinal cord, C0228589 : Structure of spinal cord spino-olivary tract |
| FMA | NCI | SNOMED CT | C1519456 : Tract of spinal cord | C1282011 : Descending spinal cord tract | C0228593 : Structure of medical reticulospinal tract, C0228594 : Structure of lateral reticulospinal tract |
| HPO | SNOMED CT | NCI | C0001623 : adrenal gland hypofunction | C0405580 : adrenal cortical hypofunction | C0948387 : Secondary adrenal insufficiency |
| HPO | SNOMED CT | NCI | C0522055 : Electrocardiogram abnormal | C0344424 : Ventricular Arrhythmia by ECG finding | C1839341 : abnormal T-wave |
| HPO | NCI | SNOMED CT | C0021828 : Intestinal atresia | C0345203 : congenital atresia of large intestine | C0549173 : Congenital atresia of rectum |
| HPO | NCI | SNOMED CT | C3272802 : Hamartomatous polyposis | C0341225 : Gastric Hamartoma | C1862304 : Hamartomatous polyp of stomach |
| HPO | MEDCIN | SNOMED CT | C0020757 : Ichthyoses | C0020758 : Congenital Ichthyosis | C0079583 : Ichthyosiform Erythroderma, Congenital |

| | | | | | |
|---|---|---|---|---|---|
| UMD | NCI | SNOMED CT | C1523994 : Diagnostic, Therapeutic and Research Equipment | C0179177 : autoclave | C0183530 : Sterilization container, C0183532 : Sterilization process indicator |
| UMD | NCI | SNOMED CT | C1523994 : Diagnostic, Therapeutic and Research Equipment | C0699733 : Devices | C0024940 : Mattresses, C0034958 : Refrigerator, C0179263 : Bedpans, C0179330 : Blanket, C0179636 : Cart, device, C0181113 : Hamper, C0181620 : Hoist device, C0181909 : Mounts, C1140607 : Commodes |
| CPT | SNOMED CT | MEDCIN | C0002903 : Anasthesia procedures | C1444819 : administration of anesthesia for procedure | C2065693 : Anestesia for procedure on knee and popliteal area , C2065716 : anestesia for procedure on shoulder and axilla, C2065727 : Anestesia for procedure on upper arm and elbow, C3495531 : Anestesia for procedure of spine and spinal cord |
| CPT | SNOMED CT, NCI | MEDCIN | C0018795 : Cardiac | C0189896 : Catheterization of right heart | C0519179 : Right heart catheterization, |

| | | | | | |
|---|---|---|---|---|---|
| | | | Catheterization Procedures | | for congenital cardiac anomalies |
| CPT | SNOMED CT | MEDCIN | C0558340 : Repair of vagina | C1288276 : Vaginoplasty | C0372573 : Vaginoplasty for intersex state |
| CPT | SNOMED CT | MEDCIN | C0203777 : Radioisotope study of endocrine system | C0203779 : Radionuclide thyroid imaging study | C2094528 : nuclear thyroid carcinoma metastases imaging |
| CPT | SNOMED CT | MEDCIN | C0337438 : Glucose measurement | C0392201 : Blood glucose measurement | C0373620 : blood glucose determination by reagent strip (lab test) |
